# Revealing the strengthening contribution of stacking faults, dislocations and grain boundaries in severely deformed LPBF AlSi10Mg alloy

**DOI:** 10.1038/s41598-023-43448-5

**Published:** 2023-09-27

**Authors:** Przemysław Snopiński, Michal Kotoul, Jindřich Petruška, Stanislav Rusz, Krzysztof Żaba, Ondřej Hilšer

**Affiliations:** 1 Department, of Engineering Materials and Biomaterials, Silesian University of Technology, 18A Konarskiego Street, 44-100 Gliwice, Poland; 2 Institute of Solid Mechanics, Mechatronics and Biomechanics, Brno University of Technology, Technická 2896/2, 616 69 Brno, Czech Republic; 3 Faculty of Mechanical Engineering, VSB-TU Ostrava, 17. Listopadu 2172/15, 708 00 Ostrava-Poruba, Czech Republic; 4 Department of Metal Working and Physical Metallurgy of Non-Ferrous Metals, Faculty of Non-Ferrous Metals, AGH University of Science and Technology, Al. Mickiewicza 30, 30-059 Kraków, Poland

**Keywords:** Mechanical engineering, Materials science, Structural materials

## Abstract

In this study, microstructural features direct metal laser melted (DMLM) aluminium–silicon-magnesium (AlSi10Mg) are investigated using advanced transmission electron microscopy (TEM) and high-resolution TEM (HRTEM). The focus is on post-processing by ECAP (Equal Channel Angular Pressing) and its effects on grain refinement, stacking fault formation and dislocation accumulation. In addition, the strength enhancing role of stacking faults is for the first time quantified. The results show that ECAP can increase the yield strength from 294 to 396 MPa, while the elongation increases from 2.4% to 6%. These results show that ECAP processing offers a new approach for producing AlSi10Mg products with improved strength and ductility.

## Introduction

Grain refinement is a widely used approach for the effective strengthening of metals and alloys based on the Hall–Petch relationship^1^. It is an inherent outcome of various physical metallurgical processes such as solidification, plastic deformation, and recrystallization^2^. However, these metallurgical processes usually result in grain sizes on the order of a few micrometers. Therefore, for the submicron or nanometer-sized grains, alternative techniques based on the severe strain accumulation are used^3^. Severe plastic deformation (SPD) is a group of metalworking techniques that involve imposing extremely high strains at relatively low temperatures^4^. These processes aim to transform bulk coarse-grained materials into ultrafine or nanograined with superior properties^5^.

In recent years, 3D-printed aluminum–silicon (Al-Si) alloys have been attracting material scientists due to their excellent mechanical and unique functional properties^6^. This group of alloys has been primarily represented by AlSi10Mg alloy, which has been the most studied alloy for DMLM, likely due to its ease of processing^7^. The microstructure of the DMLM-AlSi10Mg alloy exhibits a distinctive compositional heterogeneity that distinguishes it from its cast counterpart. It comprises fine cellular α-Al grains surrounded by a sturdy eutectic Si network^8^. This eutectic Si network serves a dual purpose: it hinders dislocation movement while increasing material strength and facilitates efficient dislocation storage. In addition, research has shown^9^ that this network is capable of inducing stacking faults during deformation, which rarely develop in deformed coarse-grained Al and its alloys since Al has a high SFE of 166 mJ/m^2^^2,10,11^.

Stacking faults (SFs) play a dual role in influencing the behaviour of glissile dislocations. On the one hand, they act as barriers, reducing the mean free path of dislocations and impeding their motion. On the other hand, SFs serve as interaction and storage sites for dislocations, facilitating their accumulation. Harnessing these effects enables the enhancement of both the strength and ductility of metallic alloys^12^.

Until now, researchers have proposed that both Hall–Petch strengthening and precipitate hardening play a leading role in the strengthening of DMLM AlSi10Mg part^13^. Among these mechanisms, the Orowan loop has been identified as the most effective^14^. Moreover, it has been demonstrated that dislocation strengthening plays an important role in improving the mechanical properties of DMLM AlSi10Mg alloy subjected to Equal Channel Angular pressing (ECAP)^15^. However, previous studies have overlooked a significant contribution of stacking faults, despite their proven significant influence on the mechanical properties of aluminium alloys^12,16,17^. Understanding the role of stacking fault-mediated strengthening is crucial for enhancing the mechanical properties of DMLM Al-Si alloys, especially considering the increasing interest in hybrid manufacturing technologies that combine 3D printing with plastic deformation processes^18,19^.

In this work, a DMLM AlSi10Mg alloy with a multi-level heterogeneous microstructure was plastically deformed to improve its strength. To achieve this, we developed a two-stage post-processing procedure for the DMLM alloy, including a short annealing phase followed by a single-pass Equal Channel Angular Pressing (ECAP) processing. Given the unique heterogeneous microstructure of the DMLM alloy, the short annealing phase aimed to partially remove residual stresses while maintaining heterogeneity. The ECAP processing phase at 100 °C aimed to manipulate the grain size of the alloy while maintaining the cellular Si network to preserve the considerable storage of geometrically necessary dislocations (generated to accommodate the plastic strain gradient) that contribute to the increased strain hardening.

This paper focuses on the detailed electron microscopy characterization of the microstructure and mechanical properties evaluation. Furthermore, it clarifies the relationship between the microstructure and the resulting mechanical properties, while also investigating and analysing the strengthening mechanisms of the ECAP-processed AlSi10Mg alloy.

## Materials and methodology

AlSi10Mg alloy samples were produced via the selective laser melting (SLM) method from a spherical gas atomized powder, of which the chemical composition is given in Table 1. The main SLM process parameters enabling the fabrication of dense samples are listed in Table 2.

**Table 1:** Chemical composition of the AlSi10Mg powder used in the SLM process, in wt.%.

Al	Mg	Si	Ti	Cu	Fe
87.8	0.5	10.5	0.15	0.15	0.09

**Table 2:** Selective laser melting process parameters.

Laser power, W	175
Layer thickness, mm	0.02
Laser scanning velocity, m/s	1.4

The SLM material was annealed for 9 min at 320 °C in a laboratory dryer (labelled as HT320 condition) and subsequently machined into a 14.75 × 14.75 × 60 mm rectangular cuboid shape. Before the ECAP process, the specimens were preheated and promptly inserted into the ECAP die with a 90° channel angle and an outer curvature angle of 20°. The specimens were pressed once at 100 °C (labelled as HT320E100 condition). A graphite-based lubricant was used to reduce the friction between the billet and the die walls.

**Figure 1:**
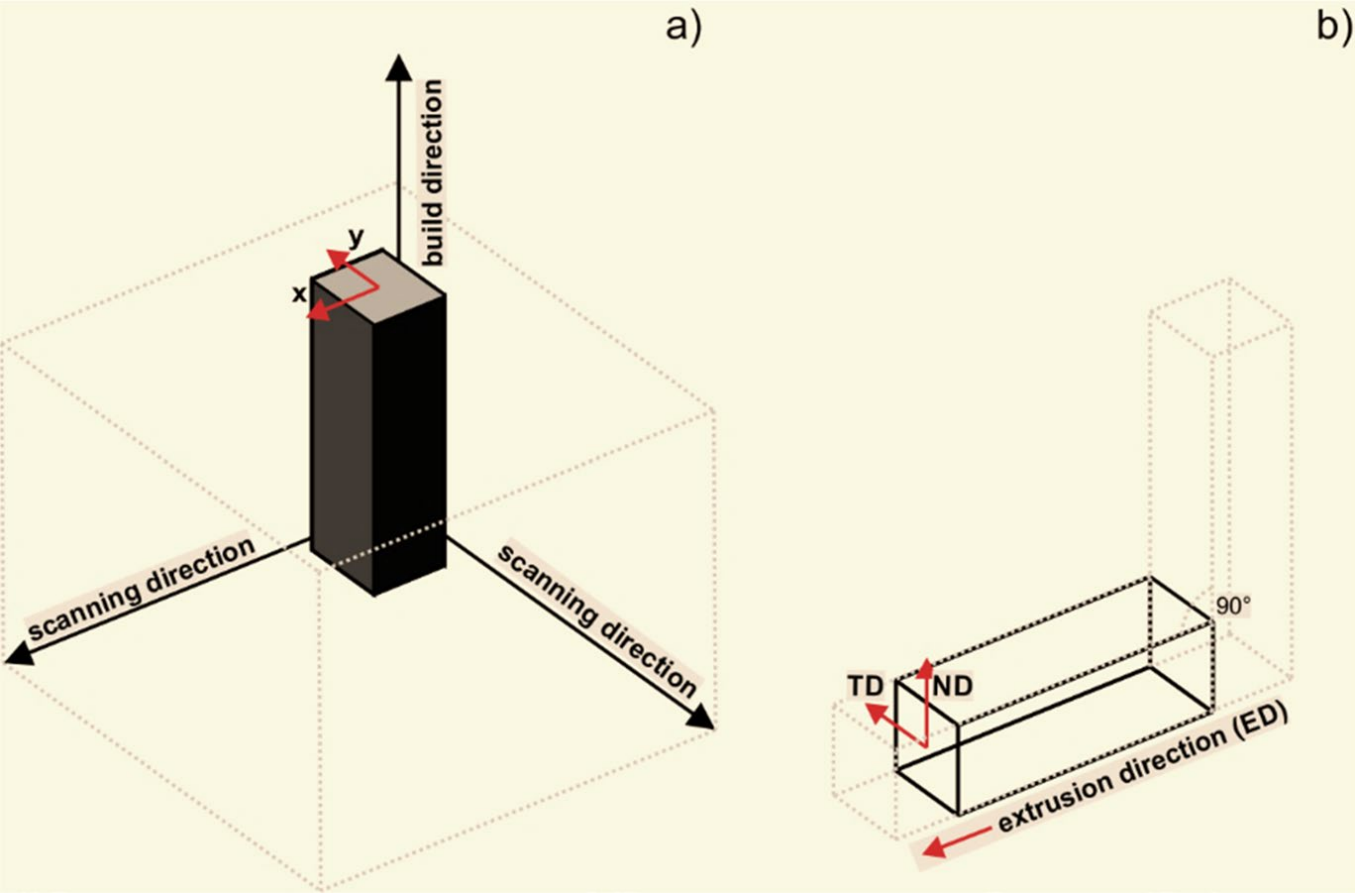
Schematic illustration of (**a)** SLM sample build direction and planes designation, (**b)** ECAP die and planes designation.

Focused Ion Beam (FIB) cutting was used to produce thin lamellas for transmission electron microscopy investigations. During FIB preparation, a lamellas were milled with Ga ions in multiple steps to achieve a final thickness of about 120 nm. The TEM lamellas were cut along the build direction (HT320) and extrusion direction (HT320E100), Fig. 1. The investigation was conducted using a Titan 80–300, FEI S/TEM microscope, capable of performing TEM, high-resolution TEM (HRTEM), and energy dispersive X-ray spectroscopy (EDS). The microscope was operated at an acceleration voltage of 300 kV. To further analyse the electron diffraction patterns, Digital Micrograph and CrysTBox (Crystallographic Toolbox) software were used.

Transmission Kikuchi diffraction (TKD) was utilized to map the crystallographic orientation of a TEM lamellae sample. This mapping was conducted on a Zeiss Supra 35 scanning electron microscope (SEM) operating at 30 kV, with a step size of 20 nm.

Crystalline size and dislocation density were determined using X-ray diffraction (XRD) analysis. The diffraction measurements were carried out within two ranges spanning 20–120°, with a step size of 0.01° and a counting interval of 5 s per step.

Tensile tests were conducted at room temperature employing a Zwick Z100 universal tensile testing machine. Sub-sized specimens were utilized for the tests, extracted in two different orientations: along the built direction (HT320 sample) and the extrusion direction (HT320E100 sample). The specimens had specific dimensions, including a diameter of 6.3 mm and a gauge length of 25.4 mm.

## Results

### EBSD results

**Figure 2:**
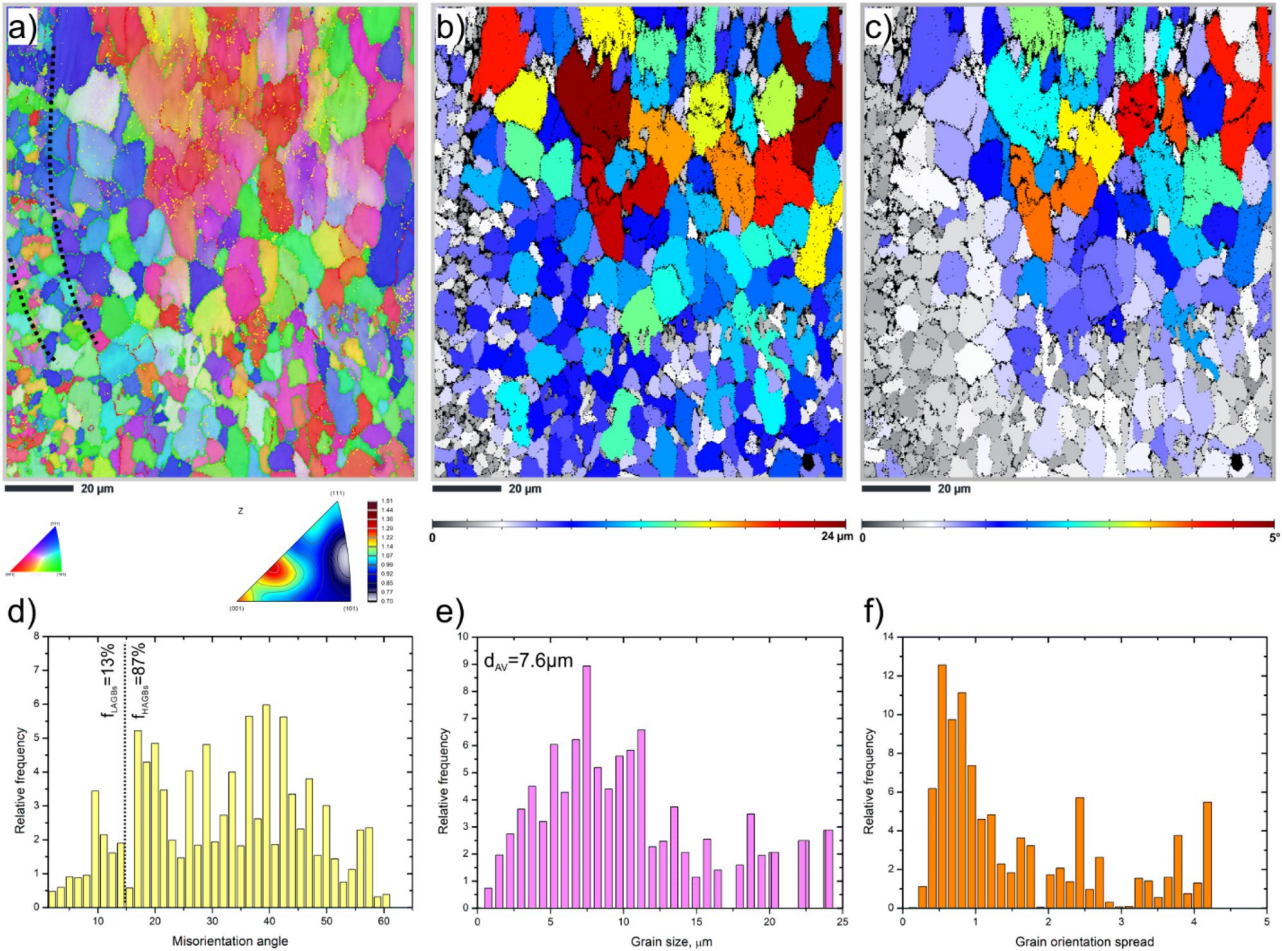
EBSD maps of the as-built HT320 sample (**a**) IPF-Z map plus grain boundaries – orange < 2ºθ < 5º, red < 5ºθ < 15º, green < 15ºθ < 65º, (**b**) grain size map, (**c**) grain orientation spread map, (**d**) grain boundary misorientation distribution, e) grain size distribution, f) grain orientation distribution.

EBSD analysis was performed to investigate the size and orientation of the grains in the as-built AlSi10Mg sample. The inverse pole figure (IPF) map, shown in Fig. 2a, reveals the presence of a fine-grained microstructure with a relatively random crystallographic texture, but with a somewhat more pronounced < 001 > textural component. In particular, the image clearly shows the boundaries of the scan traces, which are highlighted by dashed lines. According to the boundary distribution histogram, Fig. 2d, the low-angle grain boundaries (LAGBs) account for 13 ± 0.7%, while high-angle grain boundaries (HAGBs) account for 87 ± 0.9%.

The grain size map, Fig. 2b, shows clear spatial variations in the microstructure of the sample. In this figure, larger equiaxed grains can be seen within the laser scan traces. These grains exhibit a more uniform and well-defined shape, indicating a lower cooling rate. In contrast, the finer grains are mainly located at the edges of the laser scan traces. Figure 2e shows the grain size distribution histogram, which indicates that grains less than 10 µm in diameter account for about 57 ± 1.1% of the total fraction. Moreover, the average grain size is estimated to be ~ 7.6 ± 0.8 µm (GTA = 2º).

The grain orientation spread (GOS) map, as shown in Fig. 2c, reveals distinct patterns based on the GOS values assigned to different grains. The GOS of each grain is calculated considering the deviation between the average grain orientation and each point's crystal orientation within the grain. Grains exhibiting relatively low GOS values (< 2º) are indicative of recrystallized grains. In accordance with the GOS distribution histogram, Fig. 2f, it is evident that grains with GOS values < 2 account for a significant fraction, approximately 70%. Notably, these recrystallized grains are predominantly localized within the laser scan track boundaries, indicating a substantial occurrence of recrystallization in those regions. Conversely, within the laser scan track interiors, a majority of grains display higher GOS values, signifying a higher dislocation density and amount of stored energy through grain^20^.

**Figure 3:**
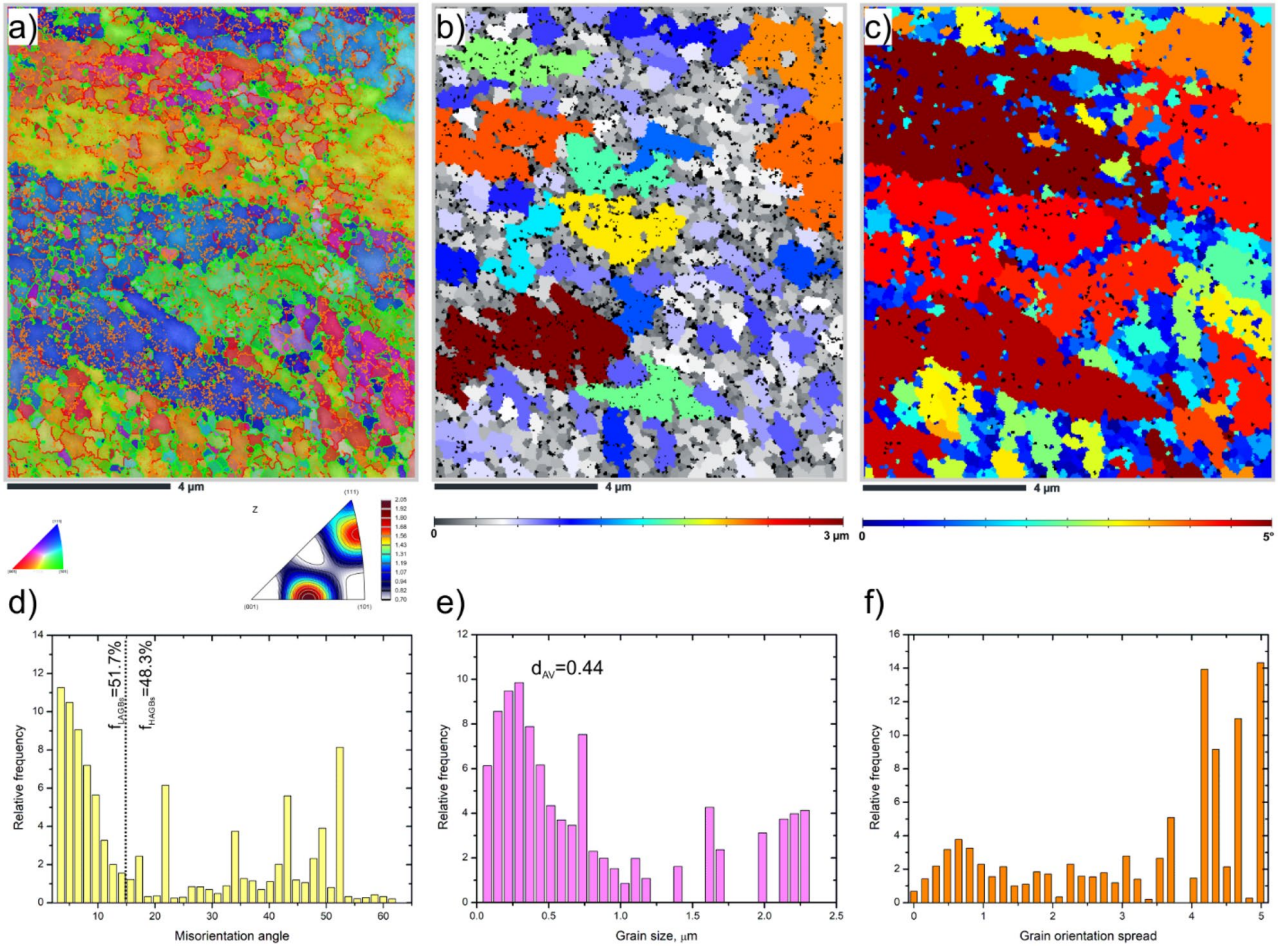
EBSD maps of the HT320E100 sample (**a**) IPF-Z map plus grain boundaries – orange < 2ºθ < 5º, red < 5ºθ < 15º, green < 15ºθ < 65º, (**b**) grain size map, (**c**) grain orientation spread map, (**d**) misorientation angle distribution, (**e**) grain size distribution, (**f**) grain orientation distribution.

Figure 3 shows the EBSD maps of the HT320E100 sample. The inverse pole figure map reveals the presence of an submicrometre microstructure with predominant < 111 > and < 101 > crystallographic orientations, Fig. 3a. Notably, the unique microstructural features resulting from the fabrication process, such as scan trace boundaries, disappeared after ECAP processing. According to the boundary distribution histogram, Fig. 3d, the fraction of low-angle grain boundaries increases significantly to about ~ 51.7 ± 1.2%, which means that high-angle grain boundaries constitute up to 48.3 ± 0.8%.

In Fig. 3b, it is evident that the ECAP processing results in a more uniform grain size. According to the grain size distribution histogram (Fig. 3e), grains with a diameter less than 1 µm constitute to approximately 73% of the total. The average grain size measured is approximately 0.44 ± 0.1 µm (GTA = 2º). This refinement in grain size is attributed to both simple shear deformation and complex strain path, ultimately leading to the accumulation of a high density of dislocations. The aluminum's high stacking fault energy (SFE) enhances the ability of cross-slip assisted dislocation motion, resulting in the formation of subgrains or dislocation walls with low misorientation angle^18^.

From Fig. 3c, it is evident that the fraction of recrystallized grains is larger in the HT320 than in the ECAP processed HT320E100 sample. The grain orientation spread (GOS) histogram indicates that 74% of subgrains exhibit “deformed” characteristics while 26% of subgrains are dynamically recrystallized (DRX) grains, Fig. 3f. This relatively large amount of orientation spread inside the larger grains is indicative of the development of a sub-grain structure^21^. This also means that dislocation slip occurs preferentially within larger grains, while in smaller grains grain boundary sliding dominates^22^.

### TEM results

**Figure 4:**
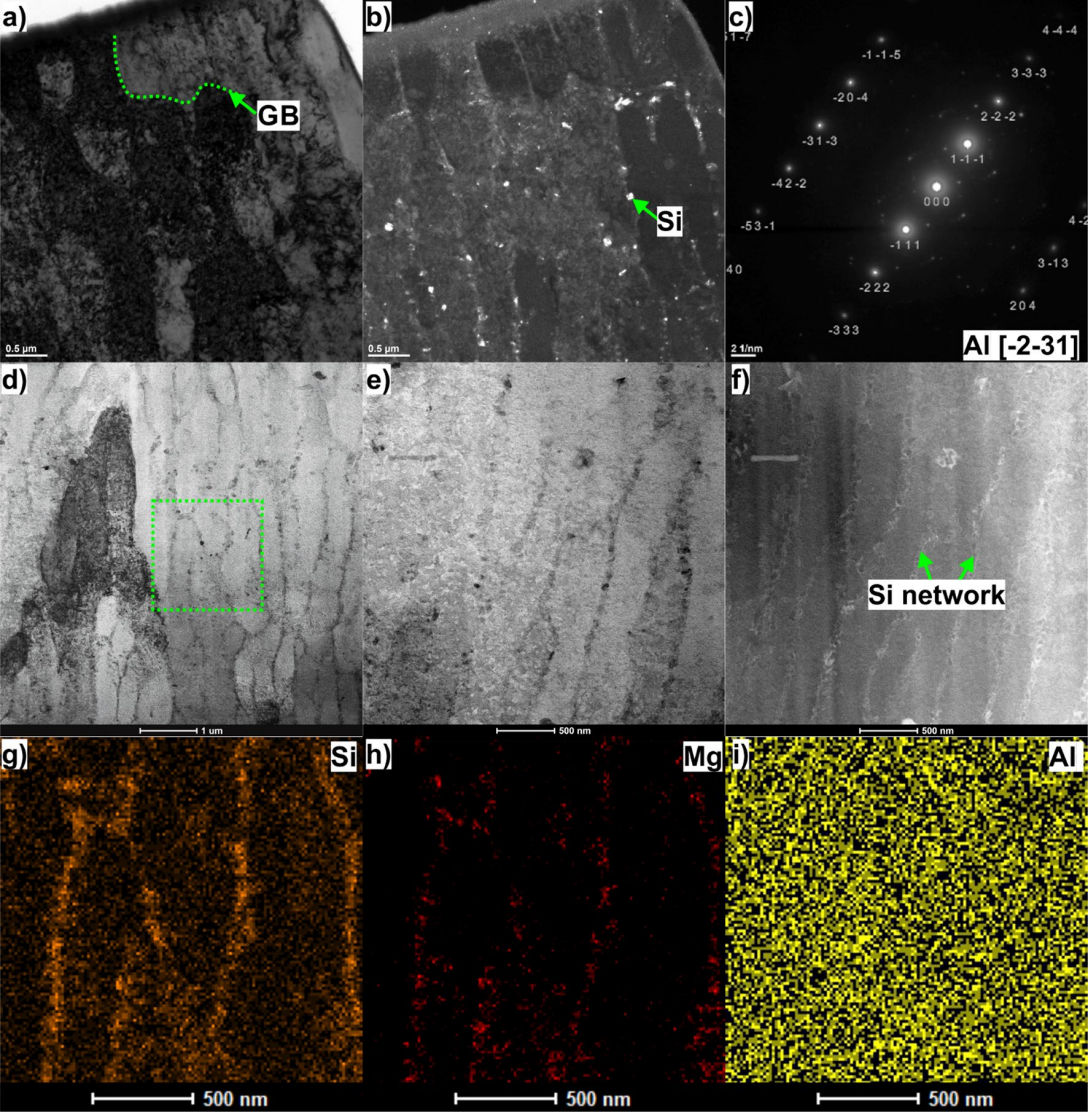
(**a**) Representative bright-field TEM micrograph of AlSi10Mg alloy in HT320 condition, (**b**) representative dark-field TEM micrograph of AlSi10Mg alloy in HT320 condition revealing Si particles at the cell boundaries, (**c**) Selected area diffraction (SAED) pattern corresponding to (**b**), (**d**) STEM image of AlSi10Mg alloy in HT320 condition revealing an area from which the EDS elemental mapping was collected (green dashed square area), (**e**) higher magnification STEM image of the HT320 sample, (**f**) corresponding STEM-HAADF image showing the net-like Si network, (**g**) Si element distribution map corresponding with the green dashed square in (**d**), (**h**) Mg element distribution map corresponding with the green dashed square in (**d**), (**i**) Al element distribution map corresponding with the green dashed square in (**d**).

Further, the TEM analyses were carried out to better understand the evolution of the microstructure of the DMLM AlSi10Mg alloy. Figure 4 shows the bright- and dark-field TEM images of the AlSi10Mg alloy under the HT320 condition. It reveals columnar cells with a high density of pre-existing dislocation networks created by the rapid cooling rate and thermal stresses during the LPBF process^23^. The columnar cell boundaries (0.5–0.8 μm wide) are decorated with randomly oriented eutectic Si particles forming a network-like structure (Fig. 4b). The corresponding EDS elemental maps (Fig. 4g–i), in combination with the SAED pattern (which consists of larger and tiny bright spots), confirm the presence of interconnected Si-rich eutectic structures.

It can be further concluded from the similar contrast observed across cell boundaries that the misorientation angle between adjacent cells is usually small, Fig. 4d–f. This statement finds support in the findings from reference^24^, which showed that adjacent cells within a single grain possessed closely matched crystallographic orientations, with misorientation angle about 0.6°. Moreover, a comparison between the cell and grain structures reveals that the cell size is at least one order of magnitude smaller than the grain boundary size (see the green dashed line in Fig. 4a).

**Figure 5:**
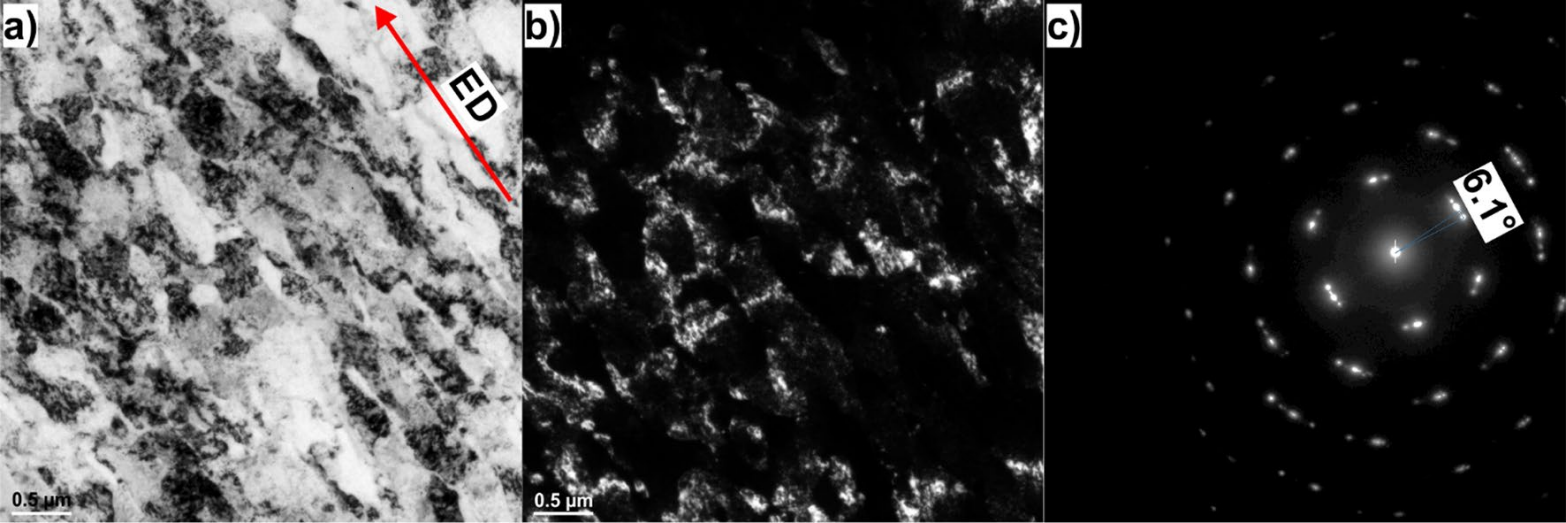
(**a**) Representative bright-field TEM micrograph of AlSi10Mg alloy in HT320E100 condition, (**b**) representative dark-field TEM micrograph of AlSi10Mg alloy in HT320E100, (**c**) selected area diffraction (SAED) pattern corresponding to (**b**). The red arrow in (**a**) marks the extrusion direction (ED).

Figure 5 shows the bright- and dark- field TEM images of the HT320E100 sample. The bright- field TEM image clearly shows the sub-microcrystalline character of the microstructure, Fig. 5a. After a single ECAP pass, the microstructure undergoes a significant alteration,which is marked by a marked increase in dislocation density and the appearance of a banded structure with boundaries that seem to have a nearly parallel orientation, Fig. 5b. The circular fringes in the SAED pattern (Fig. 5c) confirm the presence of randomly oriented nanoscale subgrains (NSGs) with low misorientation angle (as indicated by the splitting degree of SAED).

**Figure 6:**
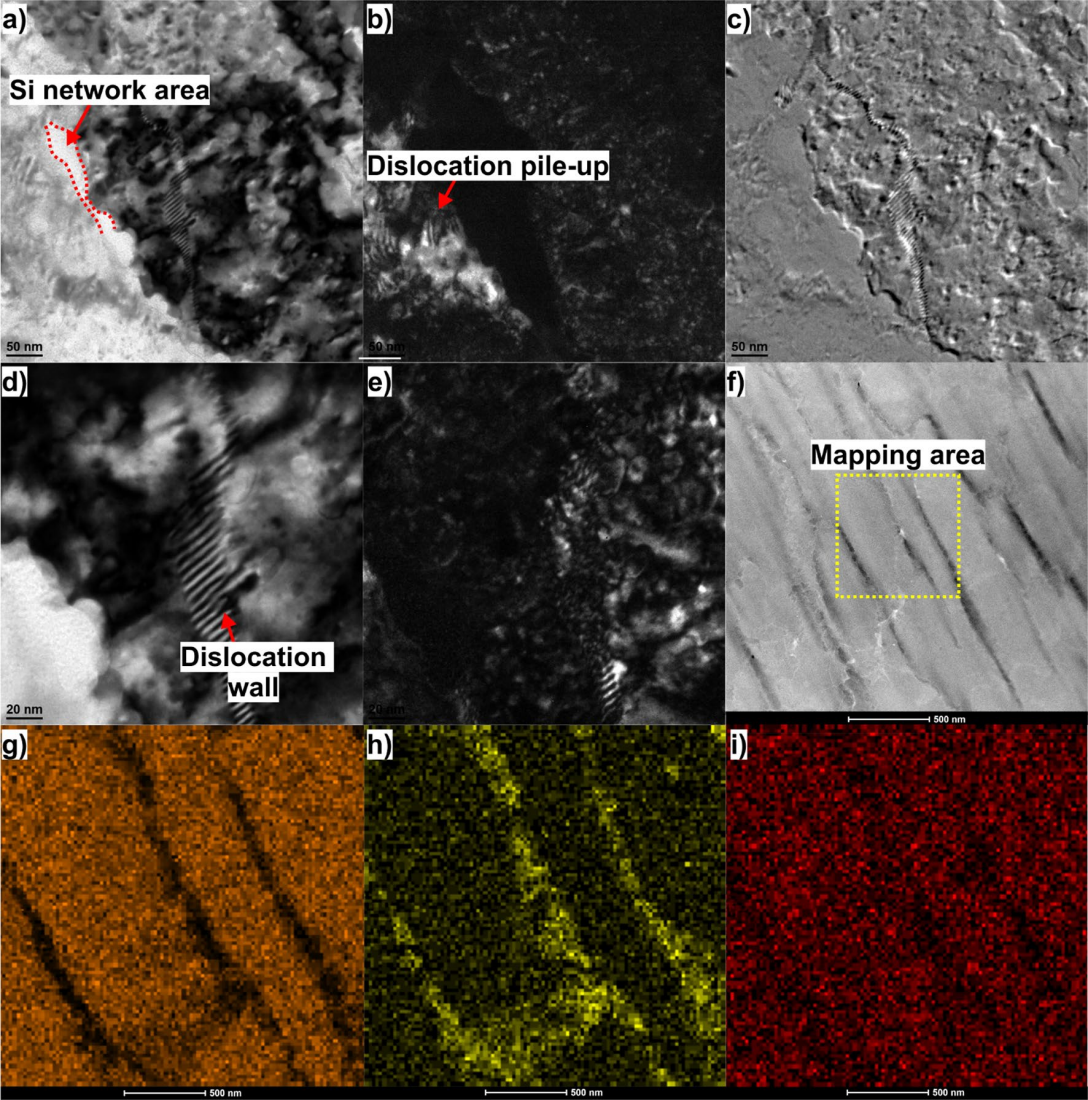
TEM analysis of the HT320E100 sample, (**a**) bright-field TEM image showing the general microstructure of the AlSi10Mg alloy, (**b**) dark-field TEM image corresponding to (**a**), (**c**) SAED of (**b**), (**d**) ) higher magnification bright field TEM image revealing the subGB region and Al-Si interface area, (e) dark-field TEM image corresponding to (**d**), (**f**) HRTEM image showing the same area as in (**d**), (**g**) STEM image in which the yellow square highlights the mapping area, (**h**), (**i**) and (**j**) distributions of Al, Si, and Mg, respectively, observed by EDS mapping.

Figure 6 shows the microstructural features of the ECAP-processed sample at higher magnification. As can be seen, the subgrain boundaries in the adjacent region of Al/Si interface (Fig. 6a) are clearly definable, indicating its higher misorientation angle. Furthermore, a substantial dislocation pile-up occurs close to the Al/Si interface area, Fig. 6b. This indicates that the Al/Si interface can effectively hinder dislocation from motion and prevent dislocation penetrating the interface, resulting in improved mechanical strength.

Closer inspection reveals a subgrain boundary formed by a dense dislocation wall (DDW), implying that LAGBs as observed by EBSD are composed of DDWs (Fig. 6d and e). These DDWs are specifically defined as geometrically necessary boundaries (GNBs), which are a type of deformation-induced dislocation boundary. This microstructure is characteristic of aluminium alloys after ECAP, where the dominant softening mechanism is dynamic recovery^25^. Referring to the STEM-BF image, Fig. 6f and Si EDS maps in Fig. 6g–i, the lamellar subgrain boundaries are mainly composed of Si, while Al and Mg are uniformly distributed within the cell interior.

**Figure 7:**
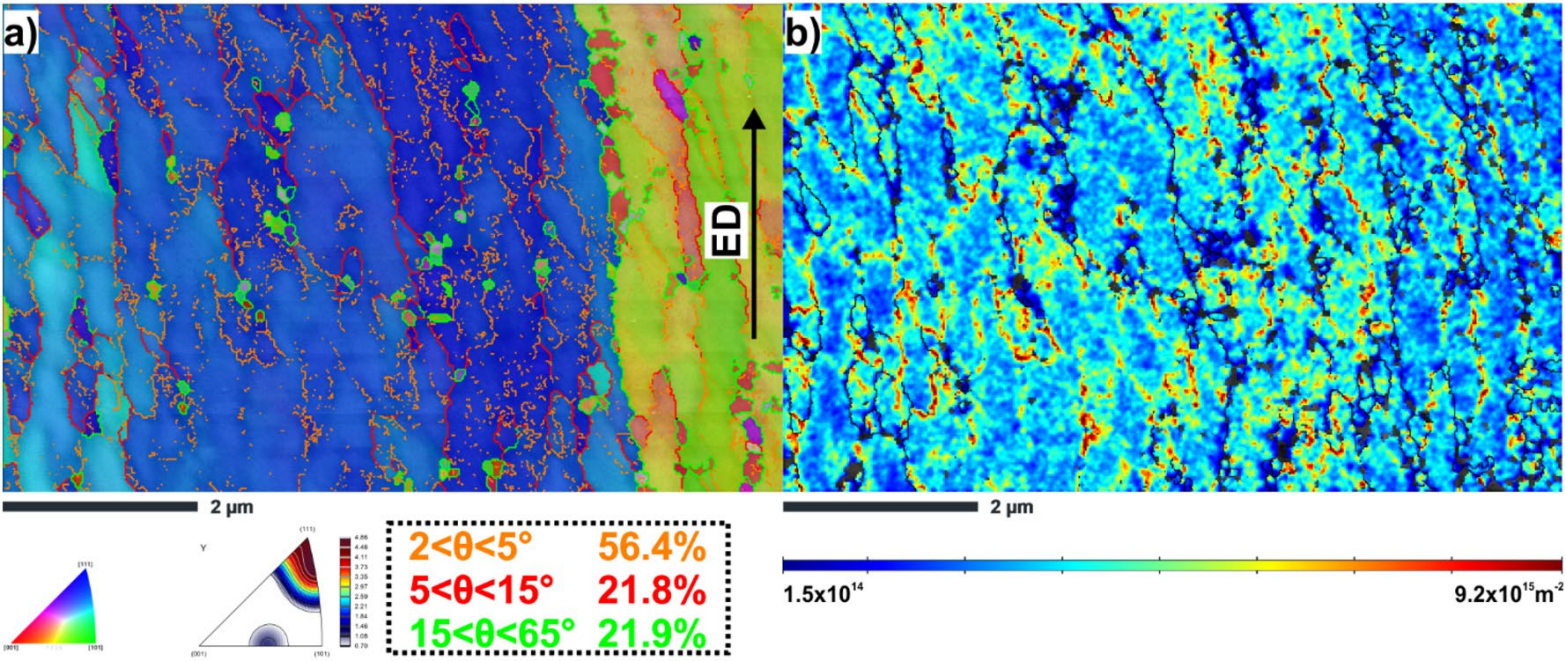
EBSD TKD maps of HT320E100 TEM lamella (**a**) IPF-Y orientation map (orange lines indicate the boundaries with a misorientation angle 2º < θ < 5º, red lines indicate the boundaries with a misorientation angle 5º < θ < 15º, green lines indicate boundaries with a misorientation angle 15º < θ < 63.5º, (**b**) GNDs distribution map calculaed on the basis of KAM values. The average GNDs density from the analysed area equals to 3.21** × **10^15^ m^−2^. The GND map confirm dislocation pile-ups arrays against interfaces (boundaries/phase boundaries). Moreover, we observe that regions of high dislocation density correlate with the gradual orientation changes seen within grains (subgrain boundaries).

Kikuchi transmission diffraction (TKD) was also used to analyse the crystallographic information of the HT320E100 TEM lamellae in detail. The IPF-Y orientation map, Fig. 7a, confirms that the microstructure consists of elongated subgrains, 200–500 nm thick, oriented parallel to the extrusion axis. The inverse pole figure, located below the IPF-Y image, reveals the predominant < 111 > crystallographic orientation in the studied area. The measured average grain size is ~ 0.32 ± 0.04 μm (GTA = 2º).

In addition, the TKD analysis shows a significant prevalence of low-angle boundaries, constituting approximately 78% of the overall boundary fraction. The occurrence of LAGBs signifies the rotation of crystal lattices, which is linked to the motion and multiplication of dislocations.

In order to evidence the grain fragmentation, the geometrically necessary distribution map (GND) is plotted, Fig. 7b. As can be seen, the positions of the GNDs interfaces correspond exactly to the positions of LAGBs, especially those with the lowest misorientation angle (2 < θ < 5º). This indicates that the GNDs transform into LAGBs during the deformation process (the dislocation walls seen in TEM images are made from GNDs).

Furthermore, based on the strain gradient theory, it is reasonable to expect a greater accumulation of geometrically necessary dislocations (GNDs) near Al/Si interfaces due to potentially significant orientation gradients around them. However, distinguishing between Al and Si via Electron Backscatter Diffraction (EBSD) proves difficult because both Al and Si lattices have cubic structures (FCC and Diamond Cubic, respectively). As a result, their Inverse Pole Figures (IPF) are recorded simultaneously, merging the microstructure of silicon with that of aluminium.

Nonetheless, it is widely reported that during plastic deformation, the strain incompatibility between these hard Si particles and the soft α-Al matrix is accommodated by the generation of GNDs. These dislocations serve as a potent driving force for the formation of substructures. In the context of the LPBF alloy, it is noteworthy that the strength of the Si cell boundary exceeds that of the FCC-α-Al matrix. As a consequence, when the alloy undergoes significant plastic strain, a local strain gradient develops near the interface. In response, GNDs are generated and accumulated at the interface to accommodate this strain gradient, ultimately giving rise to hetero-deformation-induced strengthening.

**Figure 8:**
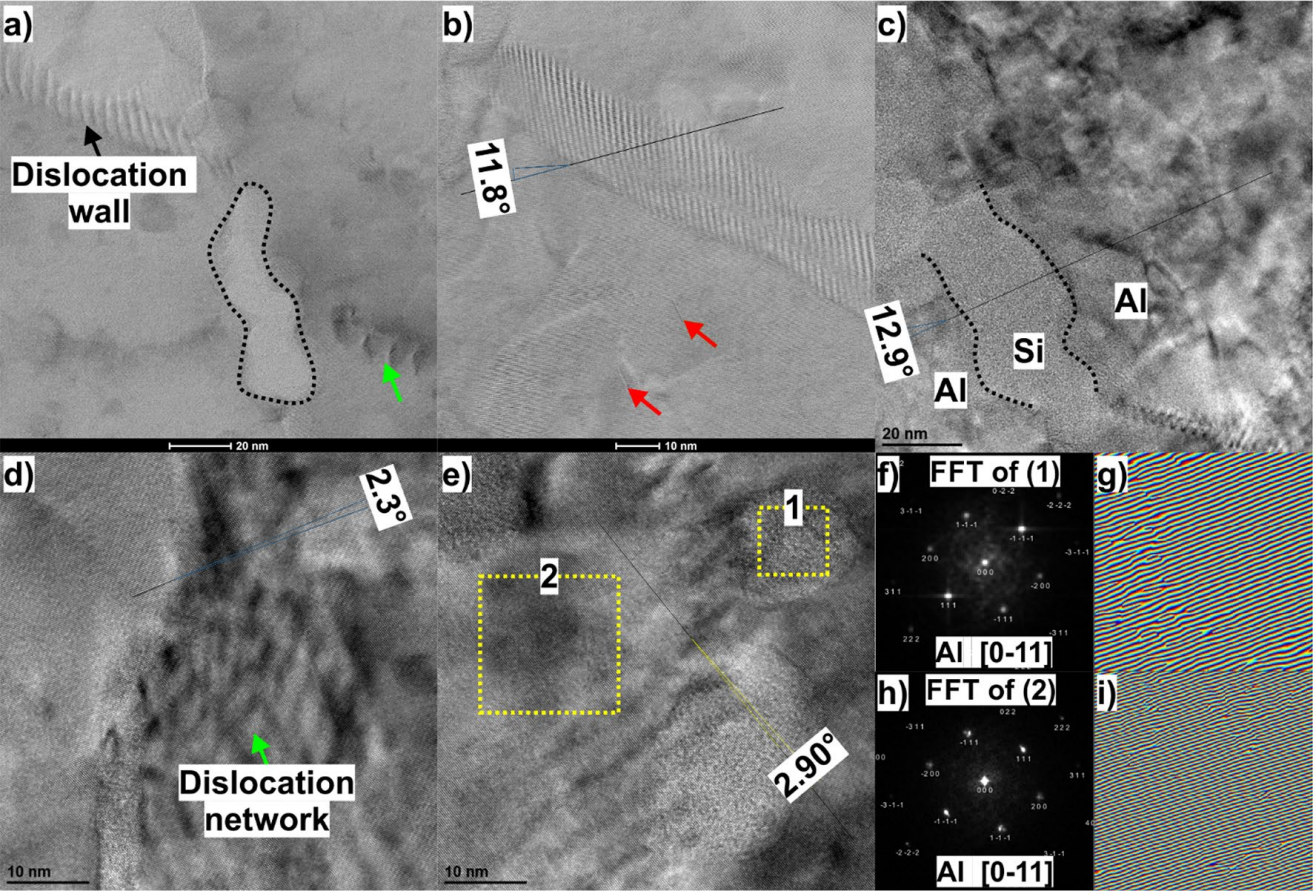
(**a**) STEM image of the Al/Si interface, (**b**) STEM image of the dislocation wall area, (**c**) HRTEM image of the Al/Si interface, (**d**) HRTEM image of the subgrain boundary formed by dislocation network, (**e**) HRTEM image of the LAGB with several sphere-like precipitates, (**f**) FFT pattern of the yellow square region #1 in (e), (**g**) raw-phase image of enlarged detail (yellow square #1) with misfit dislocations displayed, (**h**) FFT pattern of the yellow square region #2 in (**e**), (**i**) raw-phase image of enlarged detail (yellow square #2) with misfit dislocations displayed. Notably, the α-Al matrix region #2 contains fewer misfit dislocations than region #1, which correspond to the so-called “spheres”.

Further analyses were conducted in the HR-STEM and HR-TEM modes. Figure 8a shows a HRSTEM image of the Al/Si interface (the Si region is outlined with black dashed lines). It shows several dislocations that pile up at the Al/Si interface (as indicated by the green arrow). A dislocation wall separating subgrains can also be seen. A closer look at the region of the dislocation wall in Fig. 8b reveals a low-angle boundary with a measured misorientation angle of 11.8º. In addition, some stacking faults appear in the immediate vicinity of this boundary (highlighted by the red arrows).

Figure 8c shows the HRTEM image of the Al/Si interface. As can be seen, the measured misorientation at the cell boundary is about 12.9º, which indicates that the this specific urea undergoes higher plastic deformation than the cell interior. Another HRTEM image reveals the subgrain boundary formed by the dislocation network, Fig. 8d. This dislocation network compensates for a small lattice mismatch of 2.3º between the adjacent subgrains. The next HRTEM image, Fig. 8e, reveals the presence of fine spheres in the microstructure. These spheres (which may be slightly Si enriched precipitates) are fully or semicoherent and have a similar crystal structure as the surrounding Al matrix (see the FFT images in Fig. 8f–h). Additionally, geometric phase analysis (GPA) identifies several misfit dislocations in a discrete region along the interface with the matrix (Fig. 8g and i). These misfit dislocations were introduced to minimize strain and small lattice mismatch at the interface between the cluster and the alloy matrix. It should be noted that misfit dislocations can also interact with matrix dislocations, imposing an additional force on their motion. Therefore, they can contribute to the overall strength of an alloy.

**Figure 9:**
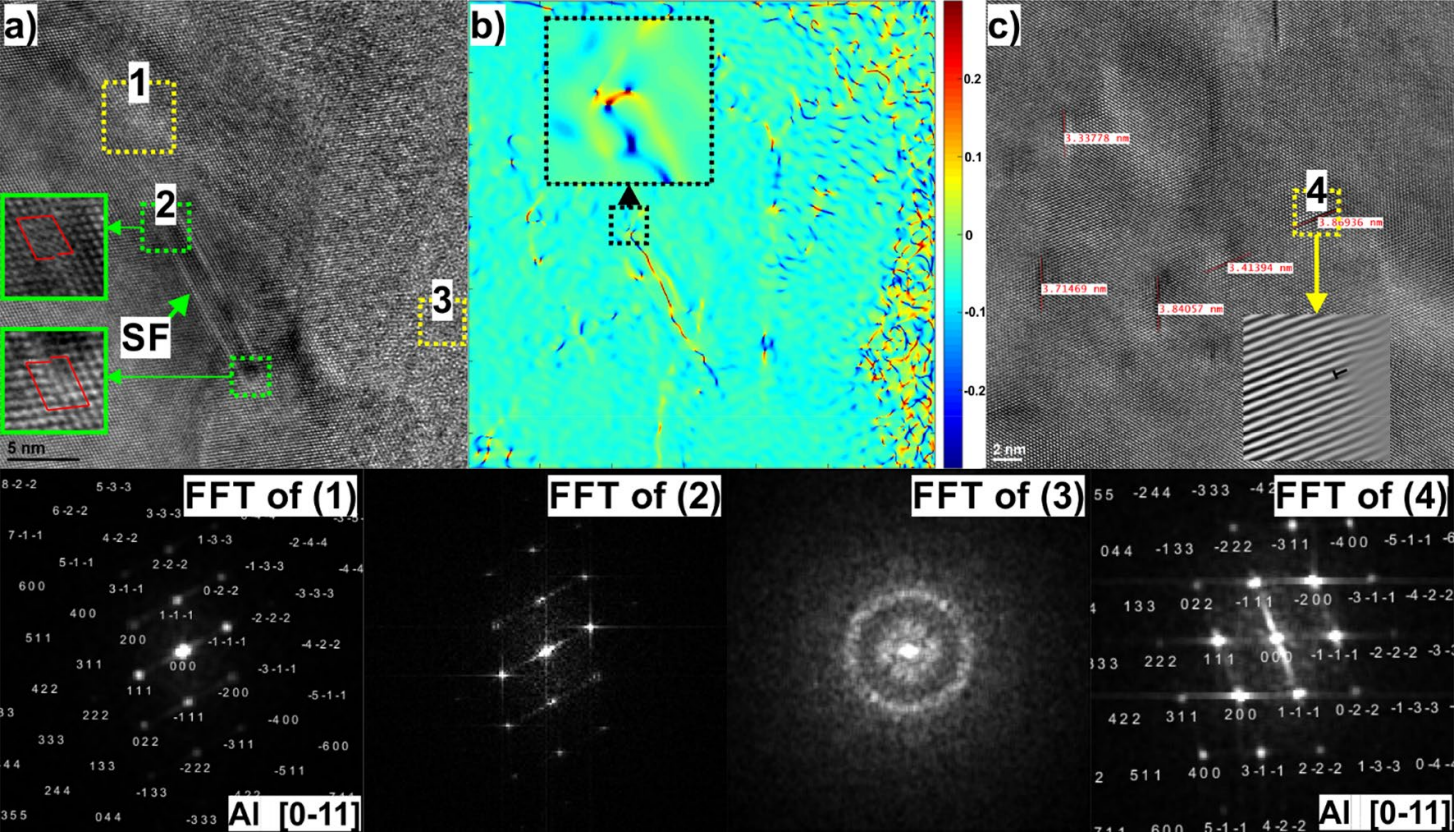
(**a**) HRTEM image taken close to the Al-Si interface (the FFT of square area #1 indicate the presence of crystalline Al phase, the FFT of square area #2 revealing streaks consistent with the diffraction shape effect of stacking faults, the FFT of square area #3 indicate the presence of an amorphous Si), (**b**) A false-colour image obtained by GPA showing the strain fields of ε_xy_. The colour scale on the right side of (**b**) illustrates the magnitude of the lattice strain, with positive values indicating tensile strain and negative values indicating compression strain. (**c**) HRTEM image of the Al matrix taken in the middle of the cell showing multiple SFs formed by Frank partial dislocations.

Figure 9 shows an HRTEM image taken near the cell boundary, providing insight into the complicated interfacial structure consisting of amorphous and crystalline Si phases. The crystalline Si layer is about 7–10 nm in thickness. Near the Al/Si interface, a stacking fault (SF) about 12 nm long can be seen. This SF was formed by the dissociation of a full lattice dislocation, which produced two Shockley partial dislocations enclosing a SF (seen in a magnified view of the green dashed regions in Fig. 9a).

The strain field surrounding the stacking fault is further analysed using geometric phase analysis, Fig. 9b. As can be seen, the strain component ε_xy_ changes from positive to negative across the stacking fault. In addition, partial Shockley dislocations causing tensile and compressive stresses (mirror symmetric) at the dislocation cores (magnified view of SF tip in Fig. 9b), are visible. What is also striking here is that the GPA shows large strain fluctuations around and within amorphous regions, suggesting that shear stresses may play a crucial role in the deformation-induced amorphization of the Si phase^26,27^. Such a large lattice distortion caused by chemical fluctuations can also act as a precipitate to pin the dislocations and produce dislocation loops, increasing the overall mechanical strength of material^28^.

It should also be noted that many SFs occur inside the α-Al face-centred cubic (FCC) cells (Fig. 8c). According to the IFFT of square area #4 in the HRTEM image (inset in Fig. 8c), these SFs are formed by Frank partial dislocations adhering to the (111) plane. In general, such SFs cause a strong obstacle to the dislocation motion and therefore can strengthen the aluminium matrix, contributing to the improvement in YS of alloy^29^.

### XRD analysis

**Figure 10:**
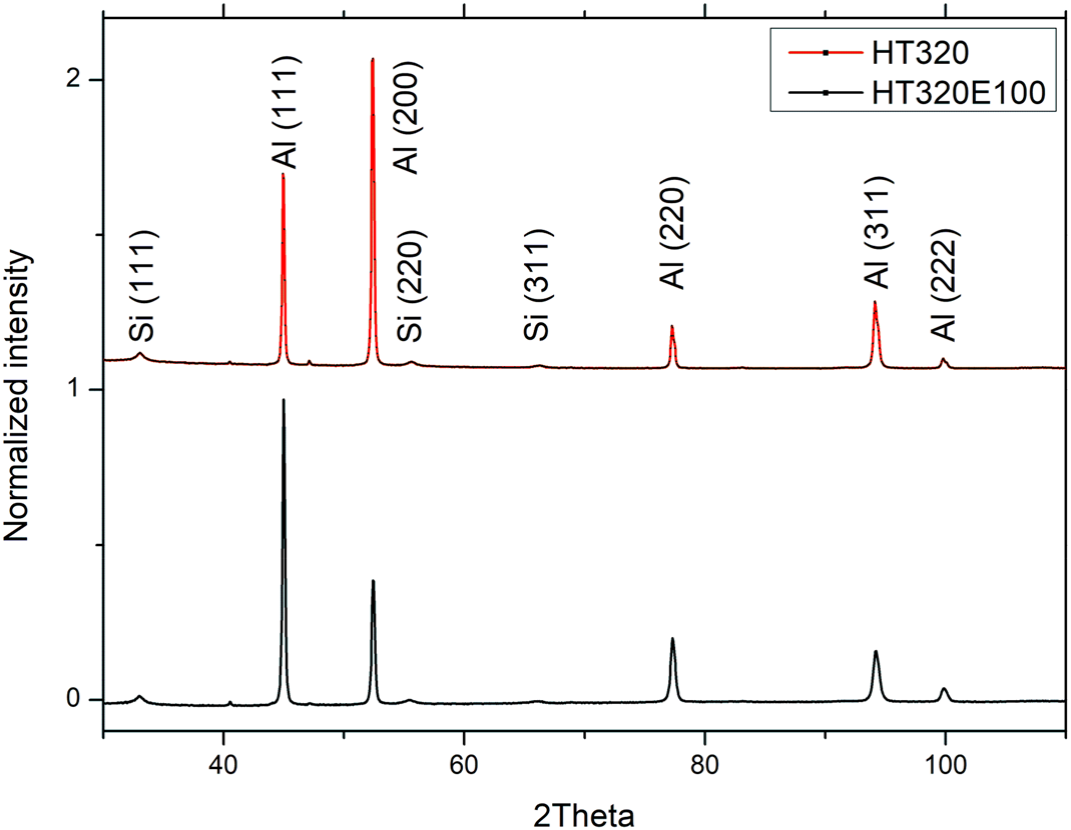
X-ray diffraction patterns of the AlSi10Mg alloy in the HT320 and HT320E100 conditions.

In Fig. 10, we present the X-ray diffraction (XRD) patterns of the AlSi10Mg alloy in HT320 and HT320E100 conditions. The diffraction patterns clearly exhibit the prominent peaks corresponding to the face-centered cubic (FCC) structure of both aluminium and silicon phases. Furthermore, a noticeable change in the peak height pattern is observed when transitioning from HT320 to HT320E100, indicating the presence of texture induced due to the Equal Channel Angular Pressing (ECAP) process. However, it is important to acknowledge that this research work does not delve into a quantitative study of the texture phenomenon. Rather, the focus of this study lies elsewhere, and any comprehensive analysis regarding the texture is beyond the scope of this particular research endeavour.

After ECAP processing, the Al lattice parameter decreased from 4.0505 Å to 4.0485 Å, which indicates a minor increase in the Si solid solubility (according to the modified Vegard's Eq. (1))^30^:

1 $$\alpha = 0.40515 - 0.0174X_{Si}$$

where α is the lattice parameter of the Al matrix, and X_Si_ is the atomic fraction of Si in Al matrix. It should be noted here that the lattice parameter equilibrium value of 4.0515 Å is typically reported for Al in AlSi10Mg alloys^31^.

Table 3 shows the main crystallographic properties, namely, the average crystallite size and dislocation density, estimated by the Williamson-Hall method. In summary, the data show that crystallite refinement occurs simultaneously with an increase in dislocation density as a result of applied deformation. According to the experimental data, the crystallite size decreases from 978 to 277 nm. At the same time, the dislocation density increases from 1.47 × 10^14^ m^-2^ to 3.46 × 10^14^ m^-2^, which is consistent with the observed vast dislocation accumulation in TEM micrographs and TKD EBSD map.

**Table 3:** Lattice parameter, crystallite size, and dislocation density values obtained through XRD analysis.

Sample	Lattice parameter, Å	Crystallite size, [nm]	Dislocation density
HT320	4.0505 ± 0.006	978 ± 14	1.47 × 10^14^ m^-2^
HT320E100	4.0485 ± 0.004	277 ± 16	3.46 × 10^14^ m^-2^

### Mechanical properties

**Figure 11:**
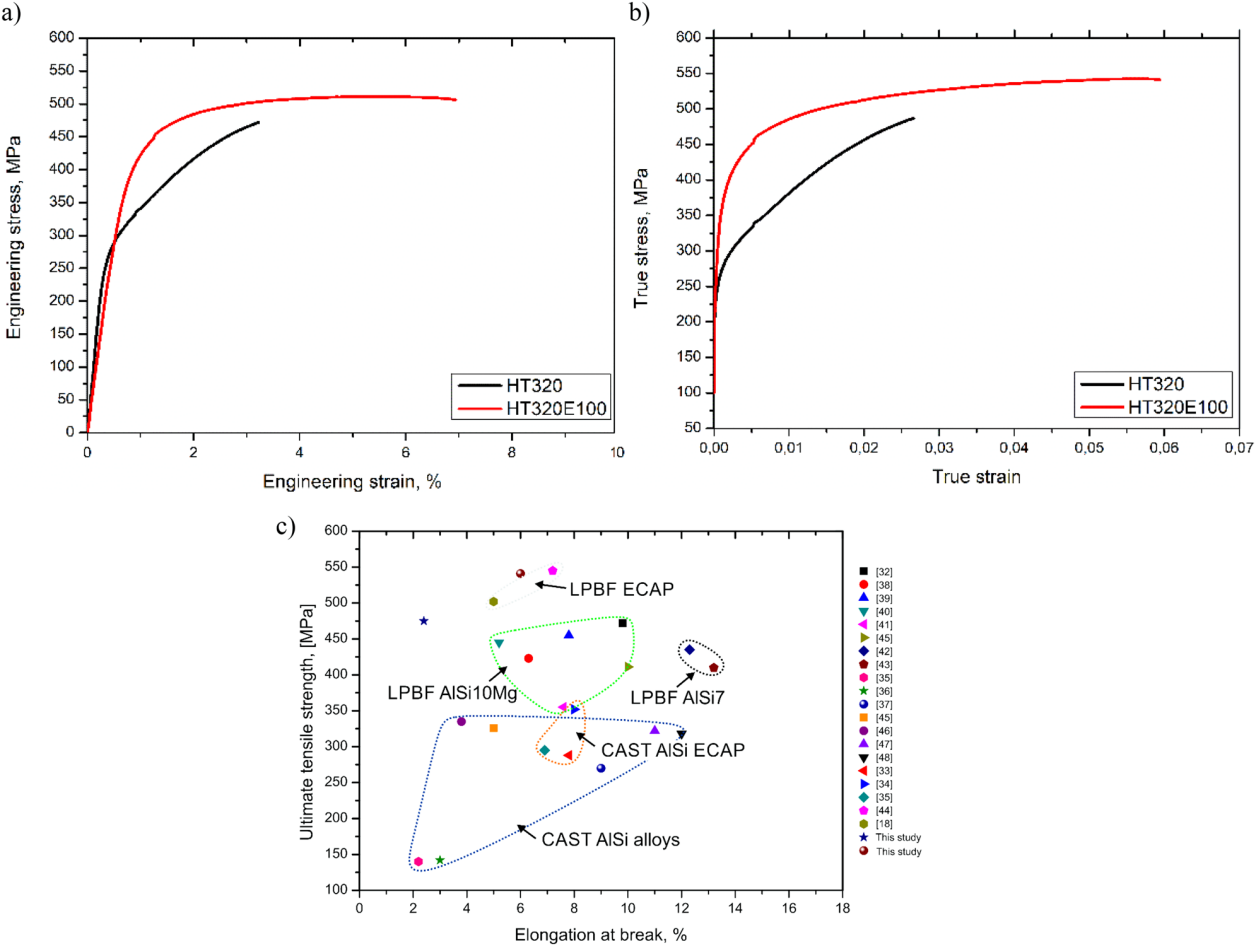
Results of the tensile tests of the AlSi10Mg alloy processed using HT320 and HT320E100 conditions. (**a**) The representative engineering stress vs. engineering strain curves and (**b**) the representative true stress vs. true strain curves of the AlSi10Mg alloy processing using HT320 and HT320E100 conditions, (**c**) comparison of UTS versus elongation at break for various Al-Si alloys, including both the current work and data from the literature^18,32–48^.

Tensile tests at room temperature were performed on HT320 and HT320E100 specimens to evaluate the mechanical properties. Figure 11a and b illustrate the stress–strain curves, while Table 4 provides a summary of key mechanical parameters. The ultimate tensile strength (UTS) of the HT320E100 sample is 541 MPa, the yield strength (YS) is 396 MPa, and the elongation is 6%. Clearly, the HT320E100 specimen outperforms the HT320 specimen in terms of strength as well as ductility. Moreover, the ultimate tensile strength (UTS) of the ECAP-processed AlSi10Mg specimens outperforms the SLM AlSi10Mg alloy with increased molten pool density (UTS ∼472 MPa)^32^ and the AlSi12 alloy subjected to four ECAP presses (UTS ~ 514 MPa)^18^. It is also superior to the cast and cast ECAP-processed Al-Si alloys, Fig. 11c.

**Table 4:** Mechanical properties of the AlSi10Mg alloy.

Condition	UTS, MPa	YS, MPa	Elongation, %
HT320	475	294	2.4
HT320E100	541	396	6.0

## Discussion

### Stacking fault formation

This research reveals the formation of considerably wide stacking faults (SFs) in the microstructure of the AlSi10Mg alloy. The presence of such wide SFs is unexpected since aluminium has a high stacking fault energy. Undoubtedly, these wide SFs can affect the mechanical properties of the studied alloy.

There are multiple hypotheses regarding the origin of stacking faults in aluminum alloys^49–51^. For instance, a Wang et al.^50^ observed a consistent correlation between the formation and growth of twins or stacking faults and the presence of stress concentrations resulting from structural heterogeneity. In our microstructural analysis, we found that the ECAP-processed LPBF AlSi10Mg alloy's deformation mechanism may differ from that of the single-phase aluminium alloy due to the presence of Al/Si heterophase interfaces. Although we were unable to detect Si using electron backscatter diffraction (EBSD), the TEM-EDS maps provided experimental evidence of Si enrichment at the lamellar boundaries.

By combining the TKD analysis (Fig. S1(b)) with the TEM-EDS elemental distribution maps, we can deduce that a high density of geometrically necessary dislocations (GNDs) emerged due to plastic strain gradients, which served to compensate for the permanent lattice curvature, particularly in the proximity of the phase and sub-grain boundaries (sub-GBs). The GNDs map thus corroborates the existence of substantial stress concentrations in these regions, which could have supplied ample stress for the initiation of twins or stacking faults (with stacking faults originating at the interface and then extending into the Al)^52^.

Alternatively, segregation of Si atoms (see Fig. 6h) near the hetero-phase interface could have reduced the local stacking fault energy (SFE), leading to an enhancement of the twinning ability of the Al matrix^53^.

The local SFE value can be calculated using the relationship given below:

2 $$\gamma_{SF} = \frac{{Gb^{2} }}{2\pi } \cdot \frac{1}{d}$$

In this equation, $\gamma_{SF}$ is the SFE per unit area, d is the average width of SF ribbon (calculated as the average SF ribbon width from Fig. 9c, d = 3.6 nm), G is the shear modulus (35 GPa), b is the length of the burgers vector (0.286 nm), and d is the average width of SF ribbon. Accordingly, this yield a SFE value of:

3 $$\gamma_{SF} = 63\frac{mJ}{{m^{2} }}$$

As can be seen, the calculated $\gamma_{SF}$ value is much lower, than the $\gamma_{SF}$ of pure Al = $123\frac{mJ}{{m^{2} }}.$ Therefore, we can hypothesise that reduced SFE may have promoted the activity of partial dislocations near the heterophase interfaces, leading to the formation of multiple stacking faults in the Al matrix (Fig. 9c). In addition, the heterophase interfaces are believed to have the potential to cause an increase in interfacial shear stress by promoting the accumulation of dislocations. This can also lead to a decrease in the effective stacking fault energy of Al, which in turn triggers the occurrence of stacking faults that can then turn into twins^54^. For example, the SFs have been observed near the Al/Ti layer interface^55^ and Al/Si interface in the Al-Si nanocomposite^56^.

It has been also postulated that the formation of high density of wide SFs is possible in nanocrystalline (NC) materials due to the emission of partial dislocations from grain boundaries^49,57,58^ as in the case of NC materials, the Frank-Read multiplication mechanism can be impeded. For example, molecular dynamics (MD) simulation^59,60^ and experimental observation^51^ have revealed that the emission of Shockley partial dislocations from GBs is activated in nanograined Al and Al alloys, generating SFs and/or deformation twins. When the grain size is close to a critical value, activation of partial dislocations is easier than that of lattice dislocations, according to Shu et al.^61^. This suggests that the formation of twin/stacking faults may be the preferred mechanism in a the ECAP-processed AlSi10Mg alloy with an average grain size of about 320 nm.

### Strengthening mechanism analysis

The microstructural characterization and quantification described above provide a solid basis for understanding the strengthening mechanisms of the ECAP-processed AM AlSi10Mg alloy. Conventional (as-cast) Al alloys are strengthened primarily by GB strengthening (the Hall–Petch relationship scales with grain size, D), dislocation strengthening, and solid solution hardening, which scales with the lattice strain field, associated with the dissolved Si. Conversely, the strength of Al-Si alloys produced by AM and subjected to ECAP processing is equal to the sum of the contributions of the Si-enriched network and the Al cell structure, as shown in Eq. (4):

4 $$\sigma_{y} = f_{cell} \left( {\sigma_{0} + \sigma_{ss} + \sigma_{disloc} + \sigma_{GB} } \right) + f_{net} \sigma_{load} + \sigma_{SF}$$

In this equation $f_{cell}$ and $f_{net}$ are the cellular and Si-enriched network fractions, respectively, and $f_{cell}$ and $f_{net}$ satisfying the equation: $f_{cell}$ + $f_{net}$ = 1. The sum of the stresses (*i.e*., $\sigma_{0} + \sigma_{ss} + \sigma_{disloc} + \sigma_{GB}$ and $\sigma_{load} , \sigma_{SF}$) represents the contribution of the friction stress ($\sigma_{0} )$, solid solution ($\sigma_{ss} )$, dislocations ($\sigma_{disloc} )$, Si-network boundaries ($\sigma_{GB} )$, stacking faults ($\sigma_{SF} )$ and the Si network load capacity ($f_{net} \sigma_{load} )$, respectively. According to Fig. S1 in supplementary file, the AlSi10Mg alloy processed using HT320E100 conditions maintains its cellular microstructure. Thus, Eq. (4) can be used to evaluate the theoretical YS.

$\sigma_{ss}$ refers to the Si and Mg solid solution strengthening effects.

5 $$\sigma_{ss} = k_{Si} (C_{Si} )^{m} + k_{Mg} (C_{Mg} )^{m}$$

where $k_{Si} and k_{Mg}$ are constants and $C_{Si} and C_{Mg}$ are concentrations of Si and Mg in solid solution (Fig. S1, Fig. S3, and Table S1 in supplementary file).

$\sigma_{disloc}$ is the strengthening effect from an increase in dislocation density after ECAP processing.

6 $$\sigma_{disloc} = \alpha MGb\sqrt {\rho_{d} }$$

where α is a material constant and ρ_SSD_ is the density of statistically stored dislocations, ρ_GND_ is the density of geometrically necessary dislocations and ρ_d_ is the sum of ρ_SSD_ and ρ_GND_, Table 5.

**Table 5:** Summarized parameters used for calculation of strengthening mechanisms.

Parameter	Description	Value
σ_0_	Friction stress, MPa	10
M	Taylor factor	2
G	Shear modulus, GPa	27
b	Length of the Burger’s vector, nm	0.284
k_d_	Constant of Hall–Petch relation for AlSi10Mg alloy, MPa*m^1/2^	0.04
d_net_	Aluminium cell size (sub-grain) size derived from EBSD, nm	320 nm
k_(Si)_	Constant for effect of Si in solid solution, wt.%^-1^	11
C_(Si)_	Si concentration in solid solution, wt.%	4.04
k_(Mg)_	Constant for effect of Mg in solid solution, wt.% ^-1^	13.8
C_(Mg)_	Mg concentration in solid solution, wt.%	0.73
m		1
α	Material constant	0.24
ρ_total_	ρ_total_ = ρ_xrd_ + ρ_GND_, m^-2^	3.55 × 10^15^
f_net_	Volume fraction of the Si-rich eutectic network	0.24
σ_load_	Load bearing of the Si-rich eutectic network at the yield point	380 GPa for Si

$\sigma_{GB}$ is the contribution of the Si-rich eutectic network impeding dislocation motion, which can be analogous to the Hall–Petch effect:

7 $$\sigma_{GB} = \frac{k}{{\sqrt {d_{net} } }}$$

where $d_{net}$ is the width of the Si-rich eutectic network (calculated as α-Al cell diameter). Upon analysing the microstructural characteristics described in this article, it becomes apparent that ECAP processing has effectively converted the Al-Si cell boundaries into subGBs. Consequently, we can consider the α-Al cell diameter as the average subgrain size, which was determined to be 0.32 µm using EBSD-TKD technique. Hence, for our calculations, we adopted a net subgrain size $d_{net}$ of 0.32 µm.

The calculated strengthening components and the corresponding YS are presented in Table 6. The theoretical $\sigma_{SF}$ strengthening effect was estimated using the following formula:

8 $$\sigma_{SF} = \sigma_{y} - \left( {f_{cell} \left( {\sigma_{0} + \sigma_{ss} + \sigma_{disloc} + \sigma_{GB} } \right) + f_{net} \sigma_{load} } \right)$$

**Table 6:** Estimated strengthening components of the HT320E100 sample in MPa.

$\sigma_{0}$	$\sigma_{ss}$	$\sigma_{disloc}$	$\sigma_{GB}$	$f_{net} \sigma_{load}$	Estimated $\sigma_{y}$ without $\sigma_{SF}$	Measured $\sigma_{y}$	$\sigma_{SF}$
10	54	220	71	91	361	396	35

It is estimated that a strength increase of about 35 MPa occurs due to the presence of stacking faults in the LPBF ECAP-processed AlSi10Mg alloy. This level of strength enhancement is easily achievable because twins and stacking faults form in materials with high SFE. For example, Sun et al.^16^ observed significant strengthening due to stacking faults in Al-CNT composites. Likewise, Lei et al.^62^ reported significant strengthening arising from deformation twins in a nano-grained Al–Mg alloy with Y-bearing prepared by mechanical alloying.

In this paper, the microstructure evolution of the DMLM AlSi10Mg alloy subjected to 1 ECAP pass at 100ºC was studied in detail. Generally, grain refinement and partial modification occurred during the ECAP process. It was also revealed that the YS of the DMLM alloy increased, which was surprisingly not accompanied by a significant loss of ductility. According to experimental observations and analysis of the strengthening mechanism, there are four main factors that contribute to the superior properties of the ECAP-processed DMLM AlSi10Mg sample. The primary mechanism responsible for the alloy’s superior properties is the dislocation strengthening. As shown above Si cell boundaries, serve as immobile obstacles within the material, effectively increasing the dislocation density. This enhanced dislocation density promotes more efficient grain refinement as the material undergoes deformation, which is another significant strengthening mechanism. Although the primary mechanism of grain refinement during ECAP involves dislocation activity^63^ and high shear deformation, the presence of non-deformable Si particles within the material cannot be underestimated. As demonstrated in Fig. 7b, the Si cell boundaries induce strain localisation and stress concentration (which is reflected by the increase in the density of GND), leading to the formation of subgrain boundaries with a higher angle of misorientation, Fig. 8c. Accordingly, with further deformation, it is expected that these subgrain boundaries with moderate misorientation angles will transform into high angle grain boundaries as a result of the dynamic recrystallisation (DRX) process.

The third is the unique heterogeneous microstructure of the alloy, which contributes to about 23% of the overall YS. Referring to Fig. S4 in the supplementary file, the cellular network was not significantly affected by ECAP shear deformation. The microstructure coarsened slightly; however, the continuity of the Si network was preserved mainly. With regard to recent literature data^64,65^, the heterogeneous cellular structure of the DMLM AlSi10Mg alloy contributes to the high heterodeformation-induced stress (HDI). This is because there is a large difference in mechanical strength between the boundaries of the soft α-Al matrix and the Si cells, approximately 9.45 GPa^66,67^. Furthermore, the Si-rich eutectic network exhibits considerably higher internal phase stress than the α-Al cell interior, thereby making a substantial contribution to the overall flow stress. Furthermore, the boundaries of Si cells (with high dislocation density and solute segregation) effectively impede dislocation movement (GNDs accumulate against the interface) and lead to a higher yield strength and strain hardening rate. It was also shown that in the case of the partially broken Si network, statistically stored dislocations can cross from one cell to another by moving through a discontinuous array of particles, which in turn permits the buildup much lower stress and improves ductility of the alloy^68^.

## Conclusions

In summary, this work demonstrates the significant ECAP-induced strengthening of AM AlSi10Mg alloys, which was achieved through the synergy of multiple strengthening mechanisms. Based on EBSD, EBSD-TKD, TEM, and HRTEM analyses, we have identified and characterized the main structural features of the ECAP processed LPBF AlSi10Mg alloy sample. The main outcomes from this research can be summarised as follows:

- Microstructural evolution of an AlSi10Mg alloy subjected to ECAP processing at 100 ºC has been presented. It is shown that the grain size decreased from ~ 7.6 µm to 0.32 µm.
- The grain refinement mechanism of the ECAP sample is mainly dynamic recrystallization.
- The microstructure of the ECAP-processed sample is primarily dominated by LAGBs, accounting for approximately 51.7% of the total. These LAGBs consist of dislocation walls and cell boundaries, with the latter exhibiting higher misorientation angles.
- ECAP improves the mechanical characteristics of the LPBF AlSi10Mg alloy. YS and UTS were improved by 112 MPA and 66 MPa, respectively, and ductility was maintained at a reasonable level of 6%.
- SFs can strengthen AlSi10Mg alloy without sacrificing plasticity. SFs contribute about 9% of the alloy yield strength.

In conclusion, ECAP shows great potential for strengthening AM materials. The experimental finding expands our knowledge of the plastic deformation mechanisms in fine-grained Al alloys, thereby contributing to a deeper understanding of how the strength of Al alloys can be enhanced through the engineering of planar defects.

### Supplementary Information

Supplementary Information.

## Data Availability

The data that support the findings of this study are available from the corresponding author upon reasonable request. Researchers interested in accessing the data for replication, verification, or further analysis can contact Przemysław Snopiński at przemyslaw.snopinski@polsl.pl.
