# Author Correction: Revealing the strengthening contribution of stacking faults, dislocations and grain boundaries in severely deformed LPBF AlSi10Mg alloy

**DOI:** 10.1038/s41598-023-44625-2

**Published:** 2023-10-18

**Authors:** Przemysław Snopiński, Michal Kotoul, Jindřich Petruška, Stanislav Rusz, Krzysztof Żaba, Ondřej Hilšer

**Affiliations:** 1 Department, of Engineering Materials and Biomaterials, Silesian University of Technology, 18A Konarskiego Street, 44-100 Gliwice, Poland; 2 Institute of Solid Mechanics, Mechatronics and Biomechanics, Brno University of Technology, Technická 2896/2, 616 69 Brno, Czech Republic; 3 Faculty of Mechanical Engineering, VSB-TU Ostrava, 17. Listopadu 2172/15, 708 00 Ostrava-Poruba, Czech Republic; 4 Department of Metal Working and Physical Metallurgy of Non-Ferrous Metals, Faculty of Non-Ferrous Metals, AGH University of Science and Technology, Al. Mickiewicza 30, 30-059 Kraków, Poland

Correction to: *Scientific Reports* 10.1038/s41598-023-43448-5, published online 27 September 2023

The original version of this Article contained an error in the Funding section.

“The Silesian Technical University, grant no: 10/010/RGH22/1125.”

now reads:

“The Silesian University of Technology, grant no: 10/010/RGH22/1125.”

The original Article has been corrected.

