# Supplementary material for: Revealing the strengthening contribution of stacking faults, dislocations and grain boundaries in severely deformed LPBF AlSi10Mg alloy
Source: Sci Rep. 2023 Sep 27;13:16166. doi: 10.1038/s41598-023-43448-5 (PMC10533813; doi:10.1038/s41598-023-43448-5)
Supplement: Supplementary file 1 — Supplementary Information. [file 41598_2023_43448_MOESM1_ESM.docx]

**Supplementary material**

Using colour threshold separation with ImageJ software, Fig. S1, we were able to reveal that the area fraction of the Si-rich phase is approximately 26.52%, with the remaining 73.48% corresponding to the primary aluminum matrix.

| **a)**  **** | **b)**  **** |
| --- | --- |

**Figure S1. (a) SEM image of the HT320E100 sample, (b) Binary image obtained using colour threshold separation with ImageJ software (white areas correspond to the aluminium cells while black to the Si cell boundaries).**.


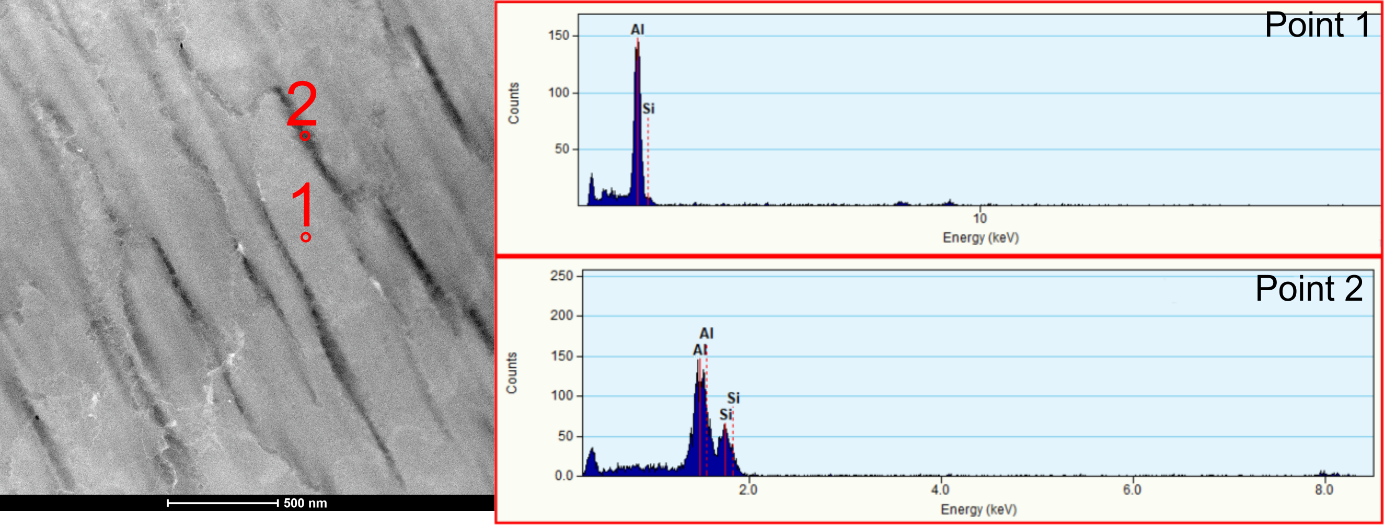


**Figure S2. STEM HAADF image of the HT320E100 alloy microstructure with a corresponding EDS spectra**

| **a)**  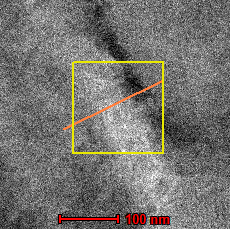 |
| --- |
| 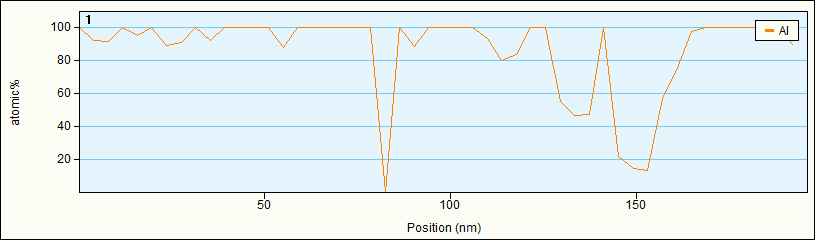 |
| 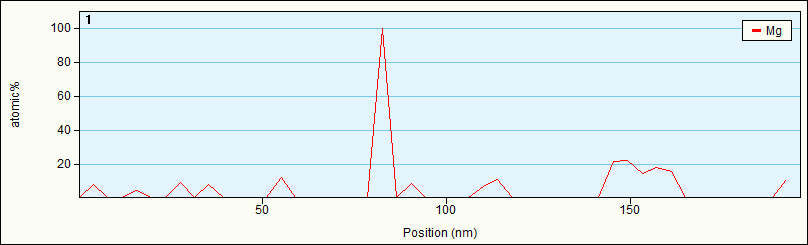 |
| **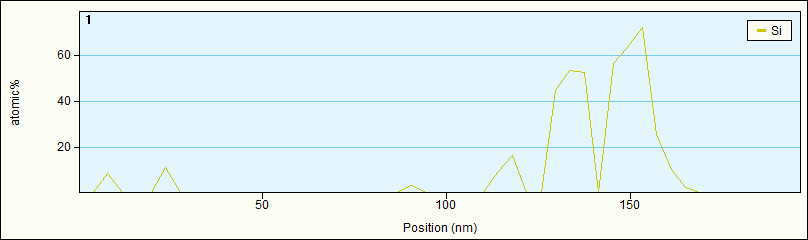** |

**Figure S3. Results of the EDS profile analysis confirming an increased Si content at the phase boundary as well as the Si content fluctuation within the cell interior**

**Table S1. Results of the pointwise chemical element microanalysis (corresponding to Fig. S2)**

|  | **Element** | **Wt.%** |
| --- | --- | --- |
| **Point 1** | Al | 57.29 |
|  | Si | 41.67 |
|  | Mg | 1.02 |
| **Point 2** | Al | 95.23 |
|  | Si | 4.04 |
|  | Mg | 0.73 |

**
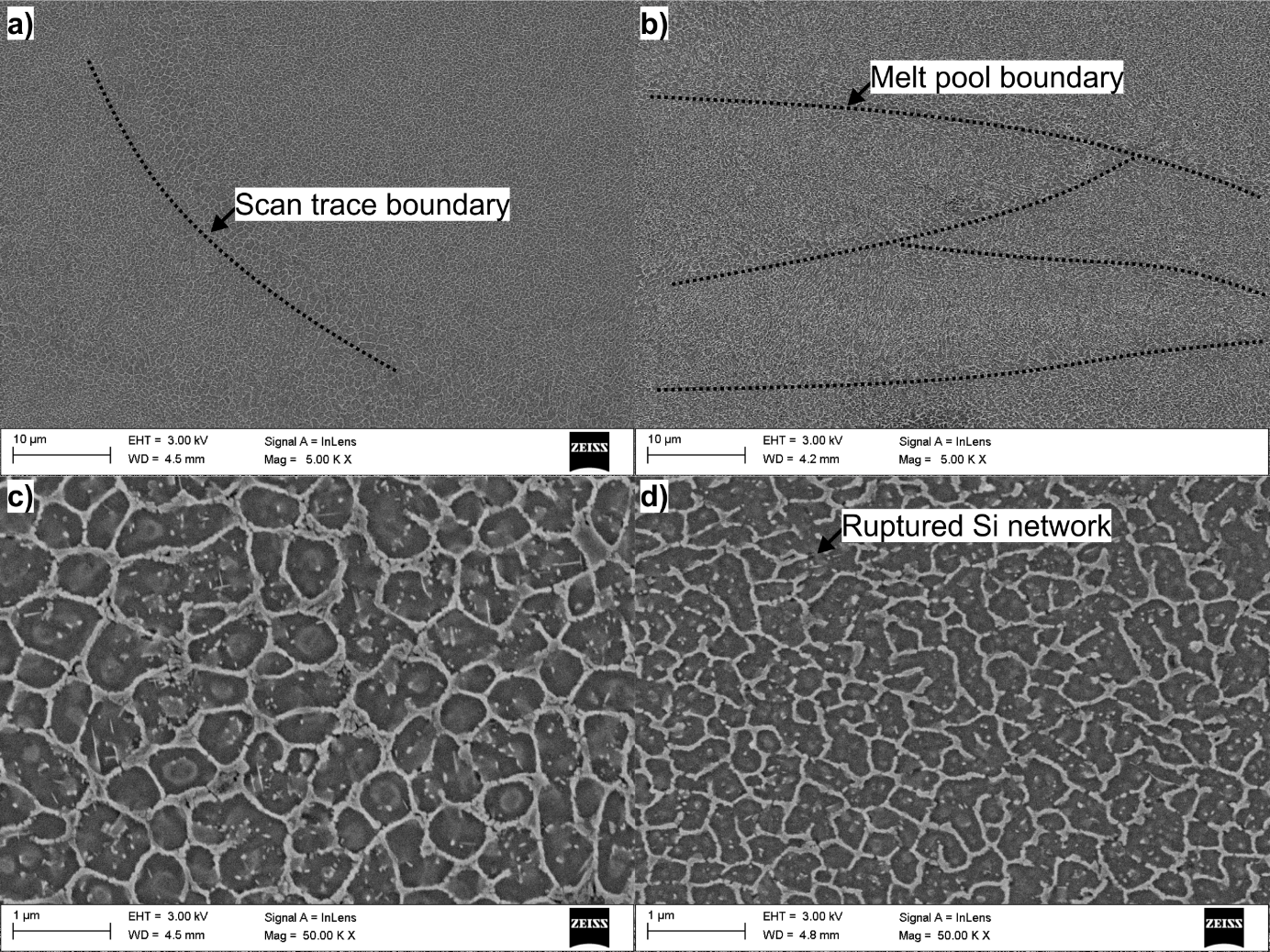
**

**Figure S4. SEM microstructures of AlSi10Mg alloy a) and c) in HT320 condition, b) and d) in HT320E100 condition.**
